# The Invisible Human-in-the-Loop: An Evolutionary Concept Analysis of Artificial Intelligence in Nursing Assistant Practice

**DOI:** 10.7759/cureus.114621

**Published:** 2026-08-16

**Authors:** Crissinee Sucharitrak

**Affiliations:** 1 Nursing, Santiago Canyon College, Orange, USA; 2 Business Analytics, California Baptist University, Riverside, USA

**Keywords:** artificial intelligence, certified nursing assistant, concept analysis, direct-care workforce, generative ai, long-term care, nursing informatics

## Abstract

Artificial intelligence (AI) is rapidly entering long-term care (LTC) environments, but its significance for the workforce performing the vast majority of hands-on care tasks, certified nursing assistants (CNAs), remains unclear. CNAs remain underrepresented in the literature on AI in nursing, which has focused on registered nurses (RNs) as clinical decision-makers. The missing element inhibits the conceptualization process and thus the research, teaching, and policymaking processes. To define this concept in the age of generative AI, this paper conducts a concept analysis using Rodgers' evolutionary methodology. The analysis is based on the nursing, LTC, and direct care technology literature. It involves 65 sources (33 empirical and 32 conceptual, methodological, governance, and policy sources) found through systematic database search and purposive interdisciplinary sampling. The four defining attributes that came up include: deployment embedded into the task as opposed to the decision; augmentation of a person who has no clinical decision-making authority; mediated agency; and dependency on interfaces that can be used by a minimally trained, low-literacy workforce. The antecedents included an unstable workforce, top-down implementation, and lack of proper training; the consequences included improved documentation but also increased surveillance, yet the influence on care quality is unknown. As an analytical contribution, the analysis develops the construct of the invisible human-in-the-loop. The concept of AI in nursing assistant practice is nascent but developing rapidly, and clarifying it now is necessary for inclusive technology design and a health informatics agenda that includes the direct-care workforce.

## Introduction and background

Who are certified nursing assistants (CNAs)?

In the United States (US), the largest group of direct care workers is made up of CNAs. There are more than 500,000 CNAs, or one-third of all nursing home workers [1,2]. CNAs perform a great part of direct care, providing bathing, dressing, feeding, and movement of clients that make up as much as 90% of direct care. Furthermore, CNAs add their input to documentation for resident care planning and help residents with physical or cognitive impairments [1,3]. However, there are large fluctuations in this workforce; high turnover rates of nurse aides can be caused by low pay, heavy workload, threat of workplace violence, and absence of career opportunities [3-7]. It is a structural problem based on the lower standards of working conditions for direct care jobs compared to the rest of the health sector workers [7,8] and changes in workforce numbers between home care and nursing homes [9]. Instability is a background against which any technology, including artificial intelligence (AI), appears.

While the literature reviewed in this paper mainly consists of research performed in high-income countries, the profession discussed is global in nature. Korean and Chinese literature on long-term care (LTC) assistants is characterized by the same pattern of high turnover intention, caused by overload, workplace violence, and poor career prospects [5,7,8]. Moreover, US assistants are increasingly immigrants, which poses additional linguistic diversity for any technology to consider [2]. Therefore, the subject of analysis is not an idiosyncratic aspect of the healthcare system of one country, but how AI integrates with front-line workers in general. Technologies described below are targeted directly at such environments.

Why should we care about AI in nursing assistant practice?

AI-based technologies have been actively used in health care since late 2022, when generative AI and large language models (LLMs) emerged [10,11]. Several umbrella review syntheses confirm the rapid spread of LLMs in clinical and administrative processes, with continuing concerns regarding accuracy, governance, and bias [12]. From the perspective of the nursing literature, there has been a series of discussions on the advantages, disadvantages, and ethical aspects of using AI [13]. LTC facilities have become places where new technologies emerge. This list includes ambient and AI-based documentation [14-16], point-of-care health information technologies (HIT) used by the nursing assistants directly [17], mobile workflow applications for care assistants [18], and assistive and service robots [19]. Ambient assisted living (AAL) and remote monitoring systems have also made their appearance in the LTC field with mixed and often limited results [20,21]. The implementation of technology is highly dependent upon the staff's acceptance [22,23]. An integrative review of implementation of HIT in the residential aged care facilities demonstrates highly heterogeneous evidence concerning the advantages of using this technology in terms of the service and quality [24]. At the same time, concept analyses and systematic reviews of clinical decision-making in nursing practice have been mostly performed within the context of registered nurses (RNs) and physicians as the clinical decision-makers [25-27]. The workforce implementing, providing, and fixing the technology remains invisible in this kind of analysis. Since CNAs do not hold clinical decision-making authority and work under the supervision of RNs, the functioning of AI in such cases cannot be the same as in professional nursing practice.

The age of generative AI adds an extra layer to the challenge above. Technologies developed in the past primarily collected data using sensors, alarms, and ambient monitoring devices. Generative AI technologies now become the core part of the care process. Transcription, drafting, and structuring of the documentation become the key functions of the technologies [14-16] and thus place AI at the core of the assistant practice, i.e., exactly in those language and literacy-related processes where a poorly trained and linguistically diverse workforce suffers from disadvantage. Conceptual clarity becomes more important than ever.

This problem can be seen as much related to the field of health informatics as to clinical practice. According to the theories of nursing informatics, the implementation of technology represents a sociotechnical process in which the competence, workflow compatibility, and organizational context determine whether the technology improves or degrades the care process [28-30]. Analysis of sociotechnical factors shows that neglecting human and organizational layers results in the silent failure of digital health systems [31,32]. The same electronic health record (EHR) systems may either decrease or increase the burden depending on the implementation [33]. Placing the CNA at the center of this framework is thus not only a matter of workforce equity but a prerequisite for digital systems that work at the point of care.

The conceptual problem

To the author's knowledge, there has not been any previous concept analysis of AI in nursing assistant/direct-care practice. While Shang [25] and Nematollahi Maleki et al. [26] have done a concept analysis on AI in nursing, the former is an older study before the advent of generative AI, while the latter is a recent one; however, both studies cover general nursing rather than the specific topic of nursing assistants and direct care practice. This gap limits training, evaluation of impacts on care quality, and policy-making. The current paper makes up for the above limitation through three main contributions: firstly, it conducts the first-ever concept analysis on AI in nursing assistant/direct-care practice; secondly, it analyzes the concept in the context of the generative AI era; thirdly, it develops the invisible human-in-the-loop construct.

## Review

Methods

In this paper, Rodgers’ evolutionary concept analysis approach was used [34]. In this approach, a concept is understood as a dynamic entity that changes with its context, and its meaning is captured through surrogate and related terms, antecedents, consequences, and attributes. The approach was chosen over the alternatives of Walker and Avant and of Morse et al. because AI use by nursing assistants is an evolving, recently transformed concept that the evolutionary approach fits best; the approach has been successfully applied in concept analyses of e-health literacy, organizational resilience, and digital health literacy [35-37].

Design and Reporting

This study is a qualitative, interpretive concept analysis. Meta-analysis and systematic review approaches have not been used because a concept analysis is not designed for quantitative synthesis of evidence. With regard to Rodgers' approach, the concept analysis included the following elements: surrogate and related terms, antecedents, defining attributes, consequences, and empirical referents for each source. The identification process was done according to the Preferred Reporting Items for Systematic Reviews and Meta-Analyses extension for Scoping Reviews (PRISMA-ScR) principles. Study parameters were defined a priori: period from January 2019 to June 2026, English language, and source type (peer-reviewed articles and organizational or governmental reports). Inferential statistical analyses were not carried out, according to the nature of the qualitative and interpretive approach. Characteristics of the sources, such as design, country of origin, and technological aspect, have been described in a descriptive way. The analysis was also conducted from a declared dual standpoint: the author of this paper is a CNA with postgraduate education in Information Technology Management and Business Analytics; to the best of the author's knowledge, it is the first concept analysis of AI in nursing done by a member of its direct-care workforce.

Information Sources and Search Strategy

The number of works on AI in CNA practice is limited. Therefore, the search for literature involved AI in nursing, AI and technology in LTC, as well as technology used by direct-care workers, following the guidelines by Rodgers that concept analysis should not be restricted to a certain field [34]. Access to bibliographic databases through programmatic means via application programming interfaces (APIs) was obtained for sources published from January 2019 until June 2026: PubMed/MEDLINE through the National Center for Biotechnology Information (NCBI) E-utilities, Europe PMC through its representational state transfer (REST) API, and OpenAlex through its API. Additional sources, including the Cumulative Index to Nursing and Allied Health Literature (CINAHL), Scopus, and Google Scholar, were reviewed for completeness. Search terms are provided in Table 1 and were combined using OR within the concept and AND between the concepts.

**Table 1:** Search strategy and information sources. Note: Concept blocks were linked using the Boolean AND operator. Bibliographic databases were searched automatically via their APIs: PubMed/MEDLINE (NCBI E-utilities), Europe PMC (REST API), and OpenAlex (API); additional databases such as CINAHL, Scopus, and Google Scholar were used for comparison purposes. The search was restricted to articles in English published from January 2019 to June 2026. AI: artificial intelligence; API: application programming interface; CINAHL: Cumulative Index to Nursing and Allied Health Literature; NCBI: National Center for Biotechnology Information; REST: Representational State Transfer

Concept block	Search terms (combined with OR within each block)
Technology	artificial intelligence OR AI OR machine learning OR deep learning OR large language model* OR generative AI OR ambient intelligence OR ambient documentation OR clinical decision support OR digital health technolog* OR health information technolog* OR assistive robot* OR service robot OR sensor* OR remote monitoring OR ambient assisted living
Worker/population	certified nursing assistant* OR nursing assistant* OR nurse aide* OR care aide* OR health care aide* OR direct care worker* OR personal care worker* OR care worker*
Setting	long-term care OR nursing home* OR residential aged care OR aged care OR care home* OR skilled nursing facilit* OR assisted living

Eligible sources included peer-reviewed articles and reports produced by organizations or governments, written in English and published from January 2019 to June 2026. The sources focused on AI or other digital health technologies in relation to nursing assistants, direct-care workers, and adjacent nursing and LTC practice. Non-English sources and sources outside the defined period, other than anchor sources, were excluded. AI in acute care physician or advanced practice settings that could not be transferred to direct-care practice was excluded. Opinion pieces without conceptual or empirical content were also excluded. Several governance and methodological sources were selected as anchor points for the framework despite their publication dates. The inclusion and exclusion criteria were defined a priori as described in the Methods.

Source Flow

The programmatic searches of three API sources yielded 623 sources (PubMed, n = 34; Europe PMC, n = 522; OpenAlex, n = 67). A total of 30 duplicates were excluded, leaving 593 articles for screening. A rule-based screening procedure was employed by identifying those articles that referenced all three concept blocks (population of direct care, digital/AI technologies, and LTC settings); each identified article was further screened manually. The inclusion criteria resulted in the selection of 33 empirical studies related to direct care, nursing assistants, and related technologies; these articles constitute the evidentiary core (Table 2). As this is an underdeveloped field, a small fraction of articles referred to CNAs as an individual occupational group; the majority of articles that have been reviewed involved direct-care and nursing staff populations including CNAs. This constitutes a finding in itself; thus, a lens-based approach was used. In accordance with Rodgers’ suggestion on an interdisciplinary nature of evidence synthesis, 32 additional conceptual, methodological, governance, and workforce-policy sources were purposively added to complement the empirical core, resulting in a final corpus of 65 sources.

**Table 2:** Characteristics of the empirical studies informing the analysis (N = 33). Note: These empirical studies center on technologies for direct care, nursing assistants, or long-term care, and they represent the core of the evidence base for the analysis. A few adjacent ambient documentation studies drawn from clinical settings are used as transferable evidence. They were combined with purposely chosen sources covering the conceptual, methodological, governance, and workforce policy perspectives to form the body of work. Only a minority of the studies focused on certified nursing assistants as a separate group, while the vast majority was concerned with larger direct care groups or nursing staff in general. AI: artificial intelligence; ED: emergency department; EHR: electronic health record; EMA: ecological momentary assessment; LLM: large language model; LTC: long-term care; RCT: randomized controlled trial; VR: virtual reality

Author (year)	Design	Country	Technology focus
Duggan MJ, et al. (2025) [15]	Mixed-methods	United States	AI ambient scribe; documentation burden and efficiency among clinicians
Guo Y, et al. (2026) [16]	Pilot mixed-methods	United States	Ambient AI documentation; efficiency, burden, patient-centered care
Meehan R (2020) [17]	User study	United States	Touch-screen point-of-care system used by nursing assistants in LTC
Miguel Cruz A, et al. (2022) [18]	Pilot mixed-methods	Canada	Mobile workflow app for health care aides; usability and acceptance
Trainum K, et al. (2024) [19]	Systematic review	United States	Nursing-staff perspectives on care robots for assisted living
Donelle L, et al. (2025) [20]	RCT	Canada	Passive remote-monitoring sensors for older home-care clients
Freeman S, et al. (2025) [21]	Mixed-methods	Canada	Under-bed sensors in LTC; resident care and staff efficiency
Iseni J, et al. (2026) [22]	Systematic review	Germany	Digital-technology acceptance among nursing staff in geriatric LTC
Kim YS, et al. (2024) [23]	Single-group pre-post	South Korea	Smart care beds tested by long-term-care workers
Bail K, et al. (2022) [24]	Integrative review	Australia	Health information technology outcomes in residential aged care
Mikkonen K, et al. (2026) [27]	Systematic review	Finland	AI supporting nurses' clinical decision-making
Kinnunen U, et al. (2023) [29]	Nationwide survey	Finland	Nurses' informatics competency in health-information-system use
Alnajjar R, et al. (2025) [30]	Cross-sectional	Jordan	Nursing-informatics perceptions of practice quality and competency
Lindsay MR and Lytle K (2022) [33]	Quality improvement	United States	EHR nursing-documentation workflow redesign (pre-post)
Lapp L, et al. (2022) [38]	Scoping review	United Kingdom	Decision-support tools for staff in adult LTC facilities
Chiu JZ and Hsieh CC (2025) [39]	Delphi study	Taiwan	Fuzzy-Delphi evaluation of an LTC nurse-aide platform
Naseer M and Dellve L (2025) [40]	Cross-sectional	Sweden	Welfare-technology use and upskilling among assistant nurses
Morris ME, et al. (2023) [41]	Mixed-methods	Australia	Digital-capability gap in the care workforce vs. technology demands
Zaman N, et al. (2021) [42]	Cross-sectional	United States	Nurses' training and perceptions of electronic documentation systems
Steven A, et al. (2019) [43]	Qualitative	United Kingdom	Hydration-monitoring app implemented by care-home staff
Omotunde M, et al. (2023) [44]	Cluster RCT (protocol)	Canada/Germany	TENA SmartCare continence indicator for nursing-home residents
Kato K, et al. (2023) [45]	User study	Japan	Transfer-support robot reducing multi-caregiver transfers in a facility
Kang K, et al. (2026) [46]	Sequential explanatory mixed-methods	South Korea	Caregivers' intention to use transfer-care robots
Wilson R and Small J (2020) [47]	Mixed-methods	Canada	Care-staff use of mobile technology for LTC communication
Rajab EA, et al. (2025) [48]	Cross-sectional	Saudi Arabia	Nurses' perceptions of electronic medical record effectiveness
Palm E, et al. (2025) [49]	Validation study	United States	Expert validation of LLM ambient-scribe clinical notes
Tan JYE, et al. (2026) [50]	Prospective observational time-motion	Singapore	Documentation time and patient interaction with an ambient AI scribe
Suzuki M, et al. (2021) [51]	Nationwide survey	Japan	Continence and pressure injury among elderly nursing-home residents
Dubois A, et al. (2021) [52]	Cross-sectional	Switzerland	Depth-sensor plus machine learning predicting fall risk in elders
Mileski M, et al. (2026) [53]	Rapid review	United States	AI applications to improve nursing-home care quality
Preiksaitis C, et al. (2026) [54]	Retrospective observational	United States	Ambient AI scribe and documentation time in the ED
Doran K, et al. (2024) [55]	Pilot mixed-methods	United States	Wearable sensors and EMA gauging LTC nursing-assistant stress
Savundranayagam MY, et al. (2026) [56]	Longitudinal qualitative	Canada	VR training for person-centered dementia communication

Data Analysis

The data sources were coded iteratively to extract surrogate terms and synonyms, antecedents, defining attributes, and consequences of the concept in question. Special attention was paid to how each of these features changed before and after the emergence of generative AI. These data were sorted and compared until a set of attributes was identified, followed by the construction of model, borderline, and contrary cases and empirical referents to analyze boundaries of the concept. In order to reduce coder bias in data analysis, the process of coding involved constant comparison, and a reflexive audit trail was established to make sure that all attributes, antecedents, and consequences were connected to the specific citations. Because the author is also a practicing CNA, reflexivity was used intentionally as a part of the methodology, and all interpretive decisions were based on scholarly evidence. Where sources differed across countries, terminologies, and worker groups, they were analyzed for conceptual rather than statistical equivalence.

Results

Surrogate and Related Terms

The concept of AI in nursing assistant practice appears in the literature under changing terminologies. These include digital health technology, health information technology, clinical decision support, ambient intelligence, information and communication technology, and assistive or social robotics [11,14,22,38]. These terminologies are used interchangeably, and in most cases, AI has been considered to fall under the digital technology umbrella rather than being mentioned as a separate concept, contributing to the conceptual confusion addressed in this analysis. The fact that these terminologies keep changing in nature and definition suggests that the concept is immature, as noted in a recent analysis of the history of digital health literacy [37]. The result is that "AI" means different things for the nursing assistant, the administrator, and the vendor. It may denote an alert threshold, a predictive model, a natural language processing model that writes a note, or a robot. Even if there is consensus on the existence of the technology, there is none on its nature. Fixing the attributes of the concept rather than its labels is what resolves these competing referents.

Antecedents

There are three recurring antecedents in CNA practice. First, the introduction of AI takes place in an environment where the organization has workforce instability: high turnover rates and staff shortages result in the use of technology to do work that cannot be done by the smaller workforce [3-7]. The administrators measure the efficiency of the technology but not how the remaining, and sometimes untrained and non-consulted, employees can be helped by it. Second, the introduction process happens on a top-down basis: the administrators and the vendors choose and configure the tool instead of the end-users, which results in acceptance happening retroactively [22,23,39]. Studies related to acceptance in geriatric LTC have found that retrospective acceptance is weak when the workforce does not participate in the design of the tool [22,39]. Third, there is an underlying assumption that the organization is digitally ready, an assumption which is not always valid. There are low levels of training: according to a survey carried out in Sweden among assistant nurses, only 52.1% were trained in welfare technology [40]. The digital gap between the care workforce and the requirements of technology-assisted care grows bigger [41], and digital health competence varies between nursing professionals [29,42]. There is also language and demographic diversity in the care workforce with different competencies [2,57]. These antecedents reinforce one another: instability prompts top-down adoption, top-down adoption bypasses training, and untrained use undermines the reliability the technology was meant to restore.

Defining Attributes

**Figure 1:**
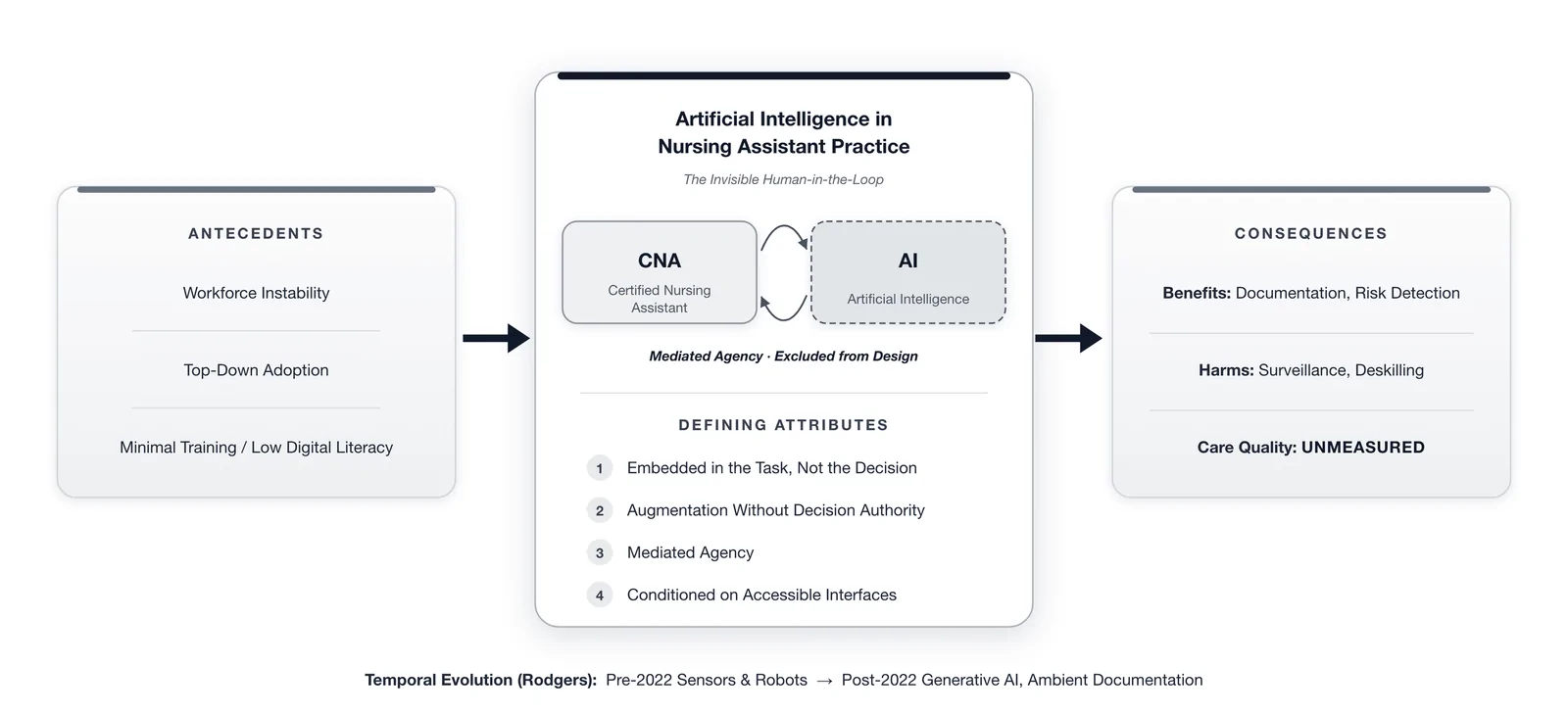
The invisible human-in-the-loop: an evolutionary concept model of AI in nursing assistant practice, showing antecedents, four defining attributes, and consequences across the emergence of generative AI. AI: artificial intelligence; CNA: certified nursing assistant

There were four attributes that distinguished the use of AI in nursing assistant practice from its use in professional nursing and that were confirmed through the review. First, AI enters CNA practice within routine, embodied tasks rather than within clinical reasoning. Examples include ambient capture during care [14-16], bedside documentation [17], mobile workflow [18], app-based hydration monitoring and sensor-based continence monitoring [43,44], and robot assistance in the physically demanding activity of patient transfer [45,46]. These are not the decision-making tools that dominate the professional nursing literature [25,26]. Second, CNAs work under supervision and are not licensed to interpret or veto the clinical interpretation provided by the system [1]. The technology thus supplements a worker who performs actions and provides data but cannot override its results. Third, the CNA possesses mediated agency: they perform the role of the human component of the system by initiating capture, providing information, and correcting mistakes [22,39] but do not participate in its design and governance. Fourth, realization of the concept depends on accessibility: the interface and documents created by the technology should correspond to the digital literacy characteristics of a minimally trained diverse workforce [28,41]; otherwise, the attributes do not manifest. Taken together, AI in nursing assistant practice may be defined as task-embedded intelligent technology that augments a supervised worker without clinical decision-making authority, whose agency within the system is mediated, and whose benefits depend on interfaces accessible to a minimally trained, linguistically diverse workforce. These relationships are shown in Figure 1.

Consequences

The consequences described and expected to be realized demonstrate a balanced profile. The positive consequences include an improved quality and efficiency of documentation and communications [14,33,47-50] and a lighter physical burden of transfers due to the use of assistive robotics [45,46]. The sensor-based continence care targets skin integrity and the pressure injury risk documented among nursing home residents [44,51], while the machine learning approach applied to continuous monitoring shows promise in early detection of risks for falling [52]. Rapid reviews of AI tools used in nursing homes show that, provided that these tools are properly deployed, they can improve care quality, although the evidence base is weak [53]. The ambient documentation literature in adjacent areas suggests possible decreases in the documentation workload for CNAs contingent upon design tailored to their literacy levels [50,54].

At the same time, these technologies broaden the scope of monitoring activities for both residents and employees. Electronic documentation was found to reduce residents' involvement in the process of care delivery and produce mixed effects on the upskilling of CNAs [40]; surveillance tools raise concerns about job insecurity and digital ageism [58,59]. More importantly, the effect of these interventions on the results of care delivery in terms of falls, pressure injuries, and hospitalization of the residents, in particular, has rarely been measured in connection with the activities of CNAs. The reviews have pointed to the lack of evidence that monitoring can predict or prevent these adverse events [24,58].

Thus, the overall effect of using the technologies on the quality of care cannot be clearly identified. Among those effects which have been quantitatively determined, mostly the positive ones were estimated in the form of gains in organizational efficiency. The negative effects which have been experienced by residents and workers were rarely measured. Thus, the tools of electronic documentation have been evaluated in terms of time saved but not the accuracy of the notes taken and the ability of the aide who created them to revise them. The monitoring systems have been assessed primarily in terms of alerts generated by them but not prevention of harm [24,58]. Such assessment is biased towards the detectability of the consequences and can distort the real picture.

Empirical Referents

The concept has observable characteristics. These are the proportion of CNA documentation generated or assisted by ambient tools, CNA responses to AI-generated alerts, use of assistive robots in patient transfers, use of mobile workflow applications [18], and representation of CNAs in technology selection and training. Recent work operationalized CNA experience directly using wearable sensors and ecological momentary assessment (EMA) to measure stress in nursing assistants during technology-assisted care [55]; thus, the concept's referents are measurable, not just theoretical. Table 3 summarizes the elements of the concept.

**Table 3:** Summary of the concept of AI in nursing assistant practice. AI: artificial intelligence; CNA: certified nursing assistant; EMA: ecological momentary assessment

Element	Content	Key sources
Surrogate and related terms	Digital health technology; health information technology; clinical decision support; ambient intelligence; information and communication technology; assistive or social robotics	[11,14,22,38]
Antecedents	(1) Workforce instability (chronic turnover, staffing shortages); (2) top-down adoption by administrators and vendors; (3) minimal training and assumed digital readiness	[2-7,22,23,29,39-42]
Defining attributes	(1) Deployment embedded in the task rather than the decision; (2) augmentation of a worker without clinical decision-making authority; (3) mediated agency; (4) realization conditional on accessible, low-literacy interfaces	[1,14-18,22,28,39,41,43-46]
Consequences (positive)	More efficient documentation and communication; reduced physical burden in transfers from assistive robotics; potential early identification of clinical risk.	[14,33,44-52]
Consequences (negative or indeterminate)	Intensified surveillance of residents and workers; job insecurity and digital ageism; effects on resident outcomes largely unmeasured.	[11,13,24,40,58,59]
Empirical referents	Proportion of documentation generated or assisted by ambient tools; responses to AI-generated alerts; use of assistive robots in transfers; use of mobile workflow applications; CNA representation in technology selection and training; sensor- and EMA-based measurement of CNA experience	[18,22,55]

Model, Borderline, and Contrary Cases

Model case: A CNA utilizes an ambient care system to document care, uses a powered transfer robot, and responds to a fall-risk warning signal. The technology that underlies this situation is obtained and configured independently of the CNA’s intervention and cannot be altered by the CNA. All four criteria are met: task embedding, absence of clinical decision authority, mediated agency, and dependence on accessibility.

Borderline case: A CNA utilizes a general-purpose chatbot on a personal device in order to collect information related to care. AI in question belongs to the domain of the CNA; however, it is not embedded into the process, and it is not approved by the organization; thus, the key criteria are partially met.

Contrary case: A CNA is involved in designing the documentation system and has the capability to overrule its decisions. This particular CNA goes through constant training in relation to virtual reality (VR) and conversational agent systems, which were developed specifically for person-centered interaction in dementia care [56]. Data-informed models of care planning based on direct-care inputs into the system likewise go beyond the existing concept [60]. These cases illustrate that the current concept is defined precisely by the absence of such conditions.

Discussion

Temporal Evolution and Conceptual Maturity

Historically, the concept itself has experienced great changes. Before 2022, discussions of technology in relation to CNA practice touched upon sensors, monitoring, and physical robotics, but the emergence of generative AI shifted the focus to the cognitive and communicative aspects of care [10-12,14]. Considering the criteria established by Rodgers, the concept under analysis is immature in its definition and boundaries but undergoes rapid development; this point provides sufficient grounds to justify its definition now. The differentiation of the current concept analysis from two earlier ones concerning AI in nursing in general comes from the perspective of the phenomenon in question: thus, Shang described AI as a source of clinical decision support for professional nurses [25], and Nematollahi Maleki et al. defined it as the enhancement of nursing care within the framework of the Walker and Avant model [26]. Current concept analysis puts the concept in a different light: that of a worker without decision-making authority and design participation. In contrast to the perspectives of professional nursing, where clinical judgement is improved, thus interpretation plays the pivotal role in analyses, from the aide's perspective, interpretation recedes, and execution, provision of data, and error correction move to the center of the concept.

The Invisible Human-in-the-Loop

The analysis leads to one conclusion: a worker placed at the operational center of AI in care, but at the periphery of decision-making and structure [22,39]. CNAs initiate, feed, and correct these technologies at the bedside while being absent from their design [22,39]; such an arrangement is herein called invisible human-in-the-loop. Earlier studies addressing the topic of AI in nursing warned of conceptual confusion, information gaps, and the necessity to develop a distinctive nursing perspective [25]; the current investigation extends these worries to the direct-care workers farthest away from the technology and clinical expertise. It fits into debates about AI governance, which implicitly presuppose the presence of a capable human-in-the-loop controlling the automated process [61,62]. In LTC, the human-in-the-loop is most often a CNA undertrained, excluded from the design, and unauthorized to override the technology. The operator is responsible for the human-in-the-loop without any corresponding power, thus undermining the safety guarantees of the "human oversight" [61,62]. CNAs are more and more working under technologies that guide, surveil, and judge their work; in other words, they are being managed by algorithms. This problem, extensively studied in the context of gig work, distributes and evaluates labor without mutual transparency, resulting in mediated agency and documented disengagement [63-65]. This new perspective on AI in LTC shifts the problem from the domain of clinical decision support to the problem of workforce inclusion and equity since direct care is mostly done by low-income and minority workers who are experiencing enhanced surveillance and digital ageism [2,58,59].

Positioning Relative to Prior Concept Analyses

The current analysis fits into a limited group of prior analyses of the concept of AI in nursing (Table 4). Shang [25], writing prior to the advent of generative AI, and Nematollahi Maleki et al. [26], writing more recently, both addressed AI in nursing in a broader sense by taking into account only the RN as an implicit clinical decision-maker, while Wangpitipanit et al. [66] studied the more focused concept of deep learning in nursing care through the lens of the Walker and Avant approach, without specifying any credentials of the working personnel. Recent systematic analysis of the available empirical evidence on AI-based interventions in nursing indicates a very similar trend at the empirical level: all of the included studies center around professional practice, education, and well-being of the nursing workforce; no studies included nursing assistants in their populations, and none were conducted in LTC facilities [67]. There are three distinguishing factors of the current analysis: it is a population-specific analysis with a focus on nursing assistants and direct-care workers, rather than nursing in general; it is concurrent with the era of generative AI and with the LTC setting; and it was done from the standpoint of an insider: the current author is at once a practicing CNA and trained in data analytics.

**Table 4:** Positioning of the present analysis relative to prior concept analyses and a recent evidence synthesis of AI in nursing. AI: artificial intelligence; CNA: certified nursing assistant; LTC: long-term care; RN: registered nurse

Analysis	Method	Population focus	Technology era	Analyst standpoint
Shang (2021) [25]	Morse’s concept analysis	Nursing broadly, with the RN as decision-maker	Pre-generative AI	Academic
Nematollahi Maleki et al. (2025) [26]	Walker and Avant	Nursing broadly, with the RN as decision-maker	Generative-AI era (by publication date)	Academic
Wangpitipanit et al. (2024) [66]	Walker and Avant	Nursing broadly; deep learning in nursing care	Machine and deep learning	Academic
Priya et al. (2025) [67]	Systematic review, not a concept analysis	Nursing practice, education, and workforce	Includes generative AI	Academic
Present analysis (2026)	Rodgers’ evolutionary	CNAs and direct-care workers in LTC	Generative-AI era	Insider: practicing CNA with data-analytics training

A Nursing Informatics Reading

Through the lens of nursing informatics, the invisible human-in-the-loop is a sociotechnical failure waiting to happen. Informatics literature maintains that a system only works where there is alignment between competency, workflow fit, and organizational setting, and that failing to take into account the human layer leads to the failure of the system [31,32]. This is a case in point: here competency is presumed, not designed [29,30]; the workflow fit is decided without involving the user [18,24], and the organizational setting is marked by instability. EHR optimization studies show that depending on the way a system is designed and trained, a system can decrease or increase burden [33,50]. In other words, an AI applied to an untrained and unconsulted group of CNAs is just as likely to create hidden work as to eliminate the existing work. This is the tangible process. An ambient system that produces notes that aides cannot check and correct transfers the burden of writing to that of checking, a displacement of burden also documented among clinicians in US settings [15,16]. If corrections are impossible, errors become silent and get entered into the record on which all decision-making depends [31,33]. Using CNAs as informatics users (i.e., measuring their competency, tailoring tools for their literacy level, providing a feedback loop from bedside to the system) is how the concept manifests itself in practice. These views represent interpretative frameworks of a scarce literature base, not conclusions drawn from it; given that very few studies isolated CNAs, the discussion needs to be seen as conceptual and research agenda-setting.

Directions for Conceptual Development

Treating the concept as emergent rather than stable provides the testable hypotheses for the next phase of inquiry. First, the involvement of CNA in technology selection and design will be positively correlated with adoption and the accuracy of the AI-assisted documentation. Second, the gap between the digital-literacy requirements of the deployed tools and the training provided will predict workarounds, alert fatigue, and disengagement. Third, the ratio of monitoring and supportive functions of a system will define whether the CNA perceives it as augmentation or as surveillance, with downstream impacts on retention. Fourth, and most importantly, the resident outcomes (falls, pressure injuries, and hospitalizations) will improve only if AI is combined with, rather than substituting, an adequately staffed and trained direct-care workforce. None of these hypotheses has been tested in relation to CNA practice, and each marks a priority for CNA-inclusive research; instruments like sensor-and-EMA stress measurement [55] and technology-acceptance surveys of care aides [18,22] show that they are measurable now. Testing them would require disaggregating nursing assistants from the broader nursing-staff category in which they are usually merged, allowing the direct-care experience to emerge in the data.

Implications

The definition of the term has certain practical consequences. When it comes to training and practice, the program should fit the real digital-literacy level of CNAs and not the assumed one, using experiential and simulation methods, which are compatible with the CNAs’ working schedule [29,40,56]. As far as design and procurement are concerned, direct-care workers should be included in the list of stakeholders, but not just end users, in order to ensure that the tool is compatible with bedside processes [18,39]. Concerning evaluation and policy, organizations should measure both resident and worker outcomes, not only documentation efficiency, and include CNAs' feedback in the organizational governance in order to make the human in the loop competent, empowered, and authorized [3,61]. Without design inclusion, training risks involving the tool designed on the basis of another workforce’s workflow; on the other hand, without outcome-focused evaluation, the organization cannot identify whether design inclusion contributes to better quality of care. Altogether, these approaches indicate that it is time to stop considering the CNA as a final point of technology decision-making and treat them as an agent of that process; all these steps are relatively inexpensive compared to the tool and are essential for the latter to function properly.

Limitations

Several limitations exist for this study. Firstly, empirical research on the application of AI within CNAs' practice is lacking. Related literature was thus reviewed regarding AI within professional nursing, technology in LTC and direct-care work, and defining attributes were deduced from that adjacent domain. The validity of these attributes is still uncertain and needs to be confirmed in future CNA-related research. Secondly, the concept is dynamic, especially in the age of generative AI, and thus the identified attributes are the defining attributes of the developing concept. Thirdly, as this concept analysis follows an interpretive methodology, it was carried out by one analyst and lacked quantitative synthesis and a risk-of-bias assessment. A reflexive approach was taken in which the identified attributes, antecedents, and consequences were grounded in cited literature; there was an audit trail, and the concept was examined via model, borderline, and contrary cases. Fourthly, the programmatic literature review was performed using open bibliographic databases (PubMed, Europe PMC, and OpenAlex), whereas subscription databases were utilized only for cross-referencing but not for systematic extraction of articles. Hence, it is possible that some articles published only in CINAHL or Scopus databases may have been missed. Fifthly, the author is a well-experienced CNA but not a professionally trained nurse or a doctoral scholar; however, this gave the author an increased sensitivity towards bedside dynamics, and all claims were grounded in the cited peer-reviewed literature. Finally, the literature search was limited to English-language sources.

## Conclusions

AI is changing LTC, but the concept of its use by CNAs, the workers who deliver the majority of direct care, is still undefined. Like the concept of AI in professional nursing, it promises much but remains immature. This analysis characterizes the concept through four defining attributes. It advances the invisible human-in-the-loop as the organizing construct linking direct-care work to nursing informatics, AI governance, and workforce equity. That construct is a necessary step toward training, evaluation, and policy that treat CNAs not as endpoints of technology decisions but as central participants in the digital transformation of care.
